# Reaction Mechanism of Oxytetracycline Degradation by Electrogenerated Reactive Chlorine: The Influence of Current Density and pH

**DOI:** 10.1021/acsomega.4c07234

**Published:** 2024-11-06

**Authors:** Stephanie Sanchez-Castrillon, Luis Norberto Benítez, Jorge Vazquez-Arenas, Franklin Ferraro, Ricardo E. Palma-Goyes

**Affiliations:** † Departamento de Química, Universidad del Valle, Calle 13 # 100-00, Santiago de Cali CP 760032, Colombia; ‡ Centro Mexicano para la Producción más Limpia, Instituto Politécnico Nacional, Av. Acueducto s/n, Col. La Laguna Ticomán, Ciudad de México 07340, Mexico; § Departamento de Ciencias Básicas, Universidad Católica Luis Amigó, Transversal, 51A, #67B 90, Medellín 050034, Colombia

## Abstract

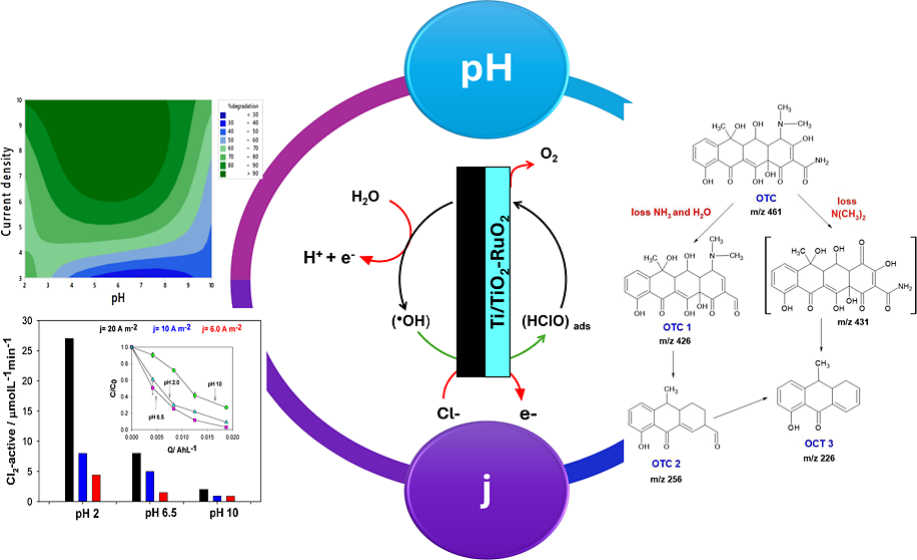

A binary dimensionally stable anode Ti/TiO_2_–RuO_2_ electrode was used to abate the antibiotic oxytetracycline (OTC) (C_22_H_24_N_2_O_9_) in chloride water. The anode was prepared using the Pechini method and subsequently characterized by X-ray diffraction, scanning electron microscopy–energy-dispersive X-ray spectroscopy (SEM–EDS), and cyclic voltammetry (CV). The optimum values of the operational parameters affecting removal efficiency were determined using a 2 × 3 factorial design by screening *j* (6.0, 10, and 20 A m^–2^) and pH (3, 6.5, and 10). The textural analysis revealed the formation of active oxides (RuO_2_ and TiO_2_ coating rutile-type *P*4_2_/*mnm*, space group 136), with a cracked surface and good dispersion of metal components. A contour graph verified that the most suitable condition for contaminant degradation was 20 A m^–2^ at a circumneutral pH of 6.5, resulting in approximately 97% degradation after 20 min of electrolysis according to pseudo-first-order reaction kinetics and the loss of the antibiotic activity of OTC. In addition, the results of oxidant formation and CV indicate that the best electrochemical activation of the anode to form Cl_2_-active mainly depended on pH. Liquid chromatography–mass spectrometry (LC–MS) and density functional theory were employed to propose a reaction pathway for OTC degradation. Three byproducts with *m*/*z* 426, 256, and 226 were identified corresponding to the removal of amide and amine groups, which are susceptible sites to electrophilic attack by active chlorine species. The findings from this work stand out for prospective applications of anodic electrochemical oxidation to efficiently eliminate antibiotics with similar chemical structures in wastewater containing chlorides.

## 1 Introduction

Tetracycline (TC) antibiotics, also referred to as TCs, are a group of pharmaceutical compounds whose main actions are bacteriostatic and bactericidal against Gram-negative and Gram-positive bacteria.^1^ Consequently, it is widely used for the treatment of cholera, typhus, and malaria and in veterinary medicine for infectious diseases of cattle and horses. OTC is one of the most used TCs used for treating the diseases described above; however, it is known that humans and animals do not completely assimilate TCs; thus, more than 50% of OTC is excreted after intake into the environment as active metabolites.^2,3^ The relative contribution of various sources of antibiotic contamination in water bodies represents a threat to human health and the environment due to the development and proliferation of antibiotic resistance genes.^4^ Additionally, antibiotic residues can serve as hot spots for the development of antibiotic resistance for microorganisms in both wastewater and treatment plants. Currently, conventional wastewater treatment methods based on chemical oxidants and biological systems are the most commonly used. However, these treatments are inefficient due to the complexity of the chemical structure (recalcitrant) and matrices (salts, organic matter, hydroxides, acids, etc.) used for treatment.^5^

In recent years, indirect electrochemical oxidation (EO) systems have emerged as alternatives for treating pharmaceutical compounds. EO by reactive chlorine species has gained attention for organic pollutant treatment in the presence of chloride ions.^6−11^ EO by reactive chlorine (Cl_2_-active) involves the electrogeneration of oxidizing agents (HClO/Cl^–^, *E*_redox_^°^ = 1.49 V vs SHE, Cl_2_/Cl^–^, *E*_redox_^°^ = 1.36 V vs SHE and OCl^–^/Cl^–^, and *E*_redox_^°^ = 0.89 V vs SHE) from chloride oxidation on a suitable dimensionally stable anode (DSA) surface, such as Ti/TiO_2_–RuO_2_, according to eqs 1–7^12,13^
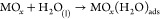
1
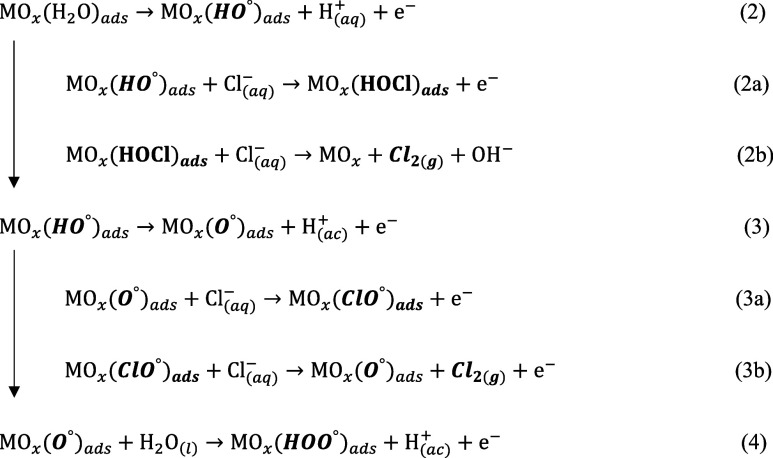
2
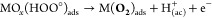
5

Chemical equilibria in solution
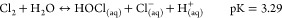
6
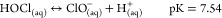
7where MO_*x*_ is the active site of the electrocatalyst surface and X_(ads)_ is an intermediate formed species on the surface of MO_*x*_. As shown in the equations above, the chlorine species on the anode surface bulk depend on the pH, electrical potential, and current density applied to the system. Accordingly, they could determine the degradation efficiency, kinetics, specific energetic consumption, and possible formation of the byproducts.

OTC was evaluated using DSAs. Qian et al. (2019) evaluated the electrochemical degradation of TCs antibiotics, such as TC, oxytetracycline, and chlortetracycline, using a Ti/SnO_2_–Sb_2_O_3_/PbO_2_ anode in 0.1 M NH_3_·H_2_O–NH_4_Cl, Na_2_SO_4_, or Na_2_HPO_4_–NaH_2_PO_4_ as a supporting electrolyte, with a maximum degradation of 82%.^14^ Ulucan-Altuntas et al. (2022)^15^ reported a hybrid system combining EO and activated carbon (AC) adsorption with removal efficiencies above 90% in the treatment of oxytetracycline from aqueous solution by a conventional one-factor-at-a-time variation approach (AC dosage, OTC concentration, and current density). They determined the effects of operating parameters on the 3D electrochemical process and conducted an optimization via fuzzy logic modeling. Belkacem et al. (2017) studied the effect of operating parameters such as chemical makeup (Na_2_SO_4_, NaNO_3_, KNO_3_, and NaCl), the dose of electrolyte support (20–100 mM), the solution pH (3–11) and the current (50–400 mA) on the removal efficiency (more than 90%) of 250 mL of OTC solution (30 mg L^–1^) using an undivided electrolytic cell under the galvanostatic mode with a Ti/Pt grid anode.^16^ However, in these reports, there is a lack of information concerning the mutual interactions of experimental variables, current density, and initial pH, which significantly affects the OTC oxidation when Ti/TiO_2_–RuO_2_ is used as an electrocatalyst and reactive chlorine species are used as oxidizing agents. Likewise, the reaction mechanism under these conditions has not been revealed. Thus, in this paper, specific efforts are devoted to optimizing the following parameters during OTC electrochemical degradation using a 2 × 3 factorial design: *j* (6.0, 10, and 20 A m^–2^), initial pH [3, circumneutral (6.5), and 10], and mutual interactions using Ti/TiO_2_–RuO_2_ prepared by the *Pechini* method. The time course of the experimental OTC concentration, evolution of oxidants formed, total organic carbon, and byproduct generation were measured to account for pollutant degradation and analyze the effects of the parameter design. The Ti/TiO_2_–RuO_2_ electrode was characterized physically and electrochemically by using X-ray diffraction (XRD), scanning electron microscopy (SEM)–energy-dispersive X-ray spectroscopy (SEM–EDS), and cyclic voltammetry (CV).

## 2 Materials and Methods

### 2.1 Electrode Preparation

Ti/TiO_2_–RuO_2_ anodes were synthesized on titanium sheets (2 × 2 cm × 0.5 mm) using the Pechini method.^17,18^ For this purpose, a solution of 4.0 g L^–1^ RuCl_3_ (a metal precursor) was added to a polymer mixture with a nominal molar ratio of citric acid (chelator) to ethylene glycol (solvent) of 1:2.4:16. The polymer precursor solution was heated to 75 °C for 30 min. The titanium sheets were previously treated with a solution of 10% oxalic acid at 80 °C for 30 min. The sheets were then washed with distilled water and acetone (Merck) for quick drying. The deposition of the catalyst layers on the sheets was carried out by immersion into the precursor solution, and the layers were subsequently placed in a muffle furnace at 180 °C for 5 min for evaporation of the solvent. This procedure was performed eight times. Finally, the sheets were calcined at 550 °C for 1 h, a temperature previously determined by TGA-DTA (Supporting Information 1). This procedure gives rise to a layer of catalyst, which was repeated a necessary number of times to obtain the anodes of the four layers of RuO_2_. The deposited mass (*w*_d_ = *w*_a_ – *w*_s_) was determined by the difference between the weight of the pretreated substrate *w*_s_ and the weight of the anode obtained at the end of the coating *w*_a_.

### 2.2 Microstructural Characterization

Morphological and microstructural analyses of the anode were performed by SEM and XRD. SEM analyses were performed using a Zeiss SUPRA 55-VP FEGSEM high-field emission microscope with an acceleration voltage of 10.9 kV. The chemical composition was determined by means of scattered-energy X-ray spectroscopy (EDS). XRD analysis was carried out using a PANalytical Empyrean diffractometer, Cu Kα, λ = 1.5406 Å, at 45 kV and 40 mA and scanned between 24 and 60° at an angle of 2θ, using the Bragg–Brentano configuration, and a PIXcel 3D detector. Matching with JCPDS-ICDD cards was used for phase and crystalline system identification.

### 2.3 Degradation Tests and Chemical and Computational Analysis

Degradation tests of 100 mL of 43.4 μmol L^–1^ oxytetracycline were performed using an undivided cell equipped with a Ti/TiO_2_–RuO_2_ anode and a titanium sheet cathode (Supporting Information 2). The pharmaceutical compound concentration was chosen considering the detection limit of the monitoring equipment used (HPLC reversal phase). The experimental tests were performed at least in duplicate.

CV studies using 0.1 mol L^–1^ NaCl and Na_2_SO_4_ for each synthesized anode were conducted with an Autolab PGSTAT 302N potentiostat-galvanostat using a scan rate of 20 mV s^–1^ and a potential range between −0.5 and +1.6 V vs Ag/AgCl in the anodic direction. Similarly, linear voltammetry (LSV) tests were performed using a scan rate of 2 mV s^–1^ and a potential range between −0.5 and +1.6 V vs Ag/AgCl in the anodic direction. A graphite rod was used as the cathode in these tests. The anode electroactive surface area was determined by the Randles–Sevcik method (eq 8)^19^ using a 4.0 × 10^–3^ mol L^–1^ ferri-ferrocyanide solution at scan rates of 10, 30, 50, 70, 90, 110, and 120 mV s^–1^.
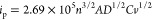
8where *i*_p_ is the peak current, *n* is the number of transferred electrons, *A* is the electroactive surface area, *D* is the diffusion coefficient of ferri-ferrocyanide, *C* is the concentration of ferri-ferrocyanide solution, and *v* is the scan rate.

The evolution of the OTC was monitored using an HPLC Hewlett-Packard 1100 series instrument equipped with an Agilent Eclipse XDB-C18 column (3.5 μm, 3.9 × 100 mm) and an ultraviolet detector operated at 360 nm. The injection volume was 20 μL. The mobile phase was oxalic acid (0.05 mol L^–1^) at 90:10 v/v under a flow rate of 1.0 mL min^–1^.

The accumulation of oxidants such as the active chlorine species electrogenerated during the treatments was determined by iodometry as reported by Serna-Galvis et al. (2021).^20^ In this analysis, aliquots of 600 μL of the sample were placed in a quartz cuvette containing 1350 μL of 0.1 mol L^–1^ KI and 50 μL of 0.01 mol L^–1^ (NH4)_6_Mo_7_O_24_·4H_2_O with subsequent stirring. The absorbance at 350 nm was measured after the reaction for 5 min in a Jasco V750 spectrophotometer.

The initial formation rate of Cl_2_-active (μmol L^–1^ min^–1^) was evaluated by linearizing the graph of the evolution of active chlorine species (in μmol L^–1^) vs the electrolysis time (min). This rate was used as an indirect indicator of the amount of oxidizing agent formed per unit of time promoted by the anode action.

To propose an EO pathway for the OTC based on molecular structure analysis, theoretical calculations of the topological and natural orbitals were carried out. Calculations were computed in the framework of density functional theory (DFT). The optimized structures (i.e., fundamental states) and frequency analyses were performed by using the B3LYP hybrid density functional. All calculations were conducted using the 6-31 + g(d) basis set, as implemented in Gaussian 16 software. The regions of the oxytetracycline molecule and its components susceptible to nucleophilic and electrophilic attack were assigned by topological analysis using the Fukui function and are represented as f^+^(r) and f^–^(r), respectively. Furthermore, potential energy surface (PES) exploration of the OTC–HOCl system was performed using the coalescence KICK method.^21^

### 2.4 Experimental Design

The 2 × 3 factorial design was conducted with the Minitab 18.0 program to assess the best conditions for pollutant degradation using active chlorine as the oxidant, and the pH and current density were varied, as shown in Table 1. The parameters of the experimental design were selected previously using preliminary studies of CV for the current density and OTC p*K*_a_s structure (p*K*_a1_ = 3.22, p*K*_a2_ = 7.46, and p*K*_a3_ = 8.94).^3^ The optimal conditions were calculated using a response contour graph based on the active chlorine generation values described in Table 1.

**Table 1:** 2 × 3 Factorial Design Used to Determine the Experimental Conditions for the Degradation of OTC Using Active Chlorine

pH	current density (A m^–2^)	degradation percentage (%)
10	6.0	49
6.5	6.0	30
2.0	6.0	80
10	10	50
6.5	10	77
2.0	10	67
10	20	73
6.5	20	97
2.0	20	91

## 3 Results and Discussion

### 3.1 XRD and SEM Characterizations

**Figure 1:**
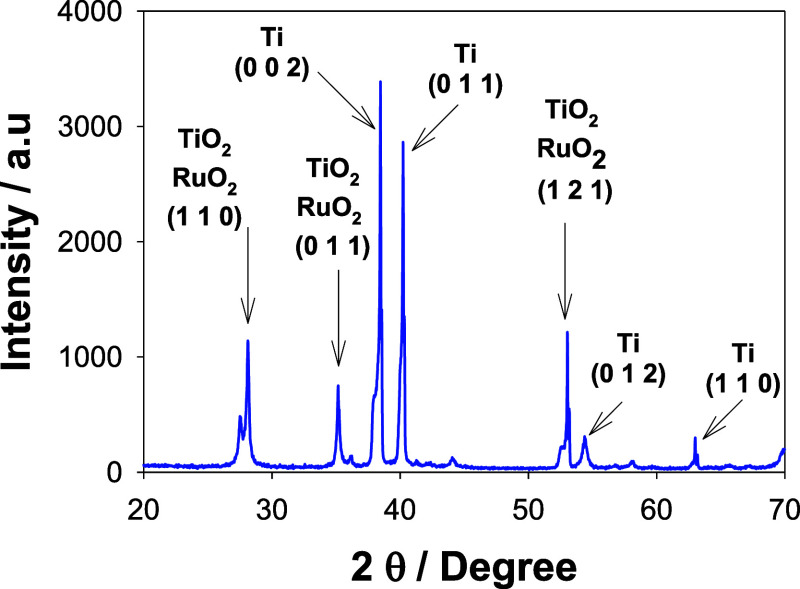
XRD pattern of the Ti/TiO_2_–RuO_2_ electrode.

Mixed oxides containing RuO_2_ are typically applied to a valve metal base, commonly titanium, in a thin-film structure to optimize the usage of precious metal components. Thermal processing is vital for enhancing the adhesion between the oxide coating and the underlying substrate and for crystallizing the active phase in the production of DSAs. Figure 1 displays the XRD patterns of the Ti/TiO_2_–RuO_2_ anodes. Matching analysis with the JCPDS-ICDD 05-1159 crystallographic card revealed the presence of RuO_2_, Ru-rich, and Ru-poor rutile-type crystalline phases (space group 136). This oxide grew on a thin layer as a mixed phase containing a small amount of anatase TiO_2_ and rutile (JCPDS-ICDD 00-9161), which was previously formed on a native Ti substrate (JCPDS-ICDD 04-3416) because of substrate pretreatment and thermal treatment during the calcination stage. Note that the predominant peaks are characteristic of tetragonal crystalline structures (*P*4_2_/*mnm*) with 2θ = 28, 35, and 54, attributed to the (1 1 0), (0 1 1), and (1 21) reflection planes, respectively. The above observation also suggested that the TiO_2_ layer could promote RuO_2_ growth, mainly because of the similar lattice parameters.

**Figure 2:**
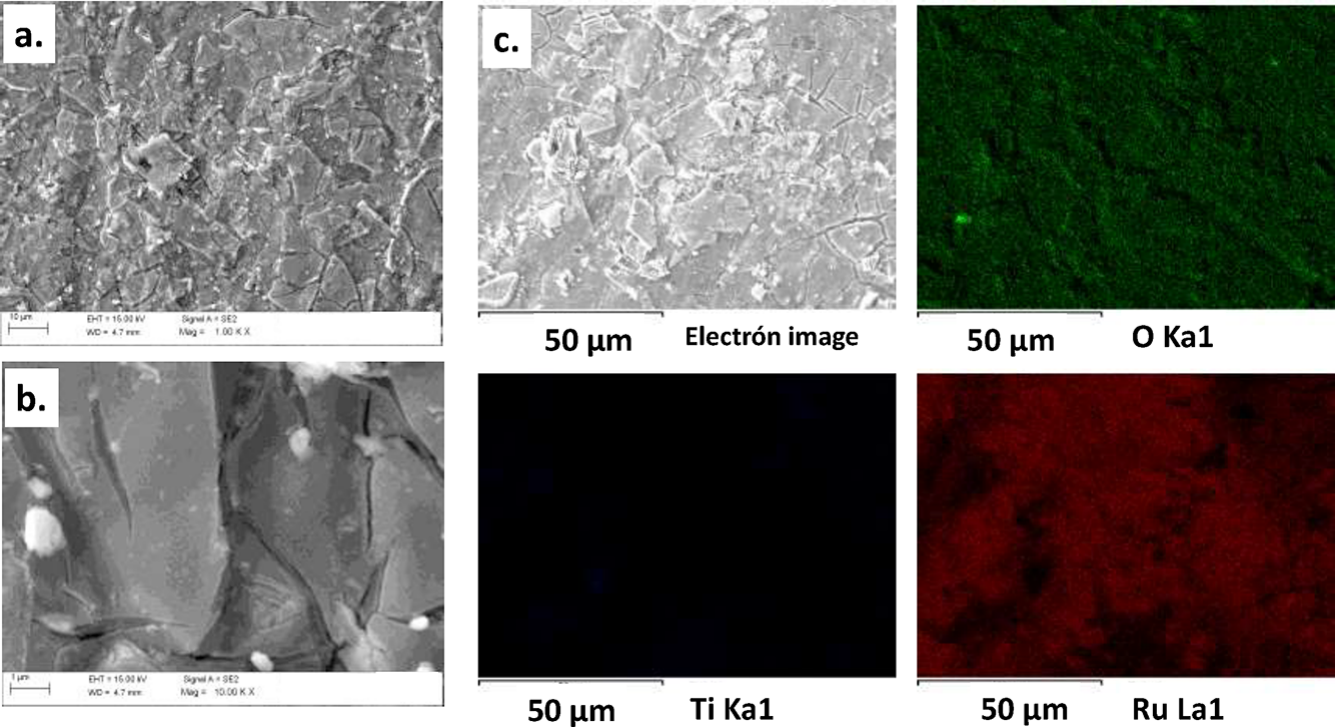
SEM–EDS images of the surfaces of Ti/TiO_2_–RuO_2_ at different magnifications at (a) 100. and (b) 10,000 K, (c) topographic image, and mapping showing the distributions of Ti, O, and Ru on the Ti/TiO_2_–RuO_2_ electrode.

Figure 2 shows SEM micrographs of the synthesized Ti/TiO_2_–RuO_2_ electrodes at different magnifications and the mapping distribution of O, Ti, and Ru on the Ti/TiO_2_–RuO_2_ electrode. As observed, the Ti/TiO_2_–RuO_2_ anode exhibited a surface conformation of mud cracks distributed along the electrode surface. These characteristics have been observed for similar oxide systems obtained by Pechini methods.^12^ EDS analyses confirmed the presence of Ru, O, and Ti (6.88 ± 1.20, 62.9 ± 3.19, and 26.4 ± 2.74 at. %, respectively). Likewise, Figure 2c shows the mapping distribution of the elements onto the anode surface, revealing a high dispersion of each along the coating.

### 3.2 Degradation Pathway, Current Density, and pH Effect on the EO of Oxytetracycline

**Figure 3:**
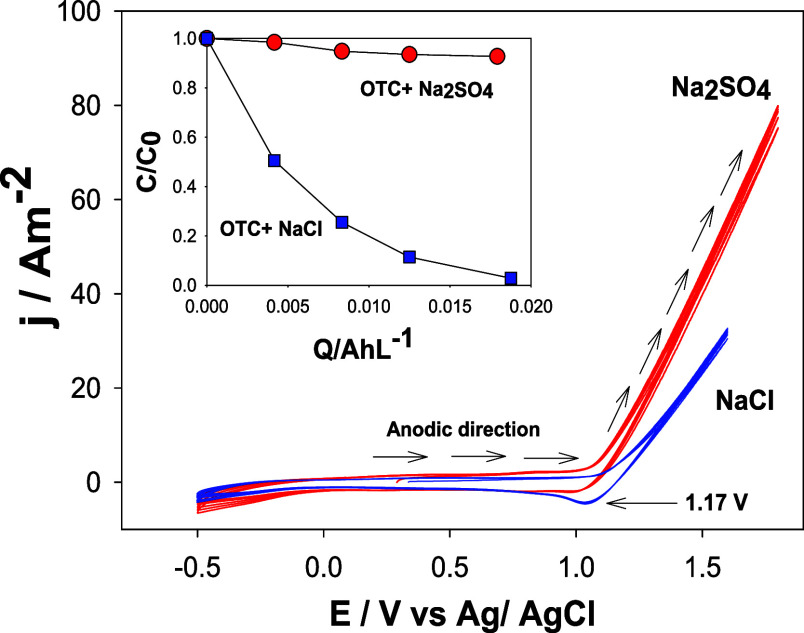
CV experiments conducted in supporting electrolytes labeled on the figure at 20 mV s^–1^ in the anodic direction (50th cycle). The inset displays the type of electrolyte effect during 43.4 μmol L^–1^ OTC degradation at 20 A m^–2^ and 0.1 mol L^–1^ electrolyte.

To determine the OTC degradation pathway, electrochemical characterizations using CV tests (Figure 3) in solutions containing 43.4 μmol L^–1^ of OTC with 0.1 mol L^–1^ Na_2_SO_4_ (line red) and NaCl (line blue) were carried out. The oxygen evolution reaction (OER) was the predominant process at 1.1 V vs Ag/AgCl (1.29 V vs SHE) in the anodic direction, without apparent signals of OTC oxidation in the scanning window when Na_2_SO_4_ was used as the supporting electrolyte. Likewise, O_2_/^•^O_2_^–^ (*E*_redox_^°^ = −0.16 V vs SHE) and O_2_/H_2_O (*E*_redox_^°^ = 1.229 V vs SHE) redox couples have not been considered in the oxidation mechanism of OTC (degradation) because they display oxidizing potentials less positive than active chlorine species; while their activities and solubilities are low in the reactor operating at 25 °C. The inset of Figure 3 shows low OTC removal after 20 min of electrolysis using 0.1 mol L^–1^ Na_2_SO_4_ and 20 A m^–2^ as the current density, ruling out direct oxidation as a priority route of degradation despite the rapid change in the oxidation slope observed via CV. On the other hand, when 0.1 mol L^–1^ NaCl was used, a cathodic signal near 1.0 V vs Ag/AgCl was evident (1.19 V vs SHE), attributed to the reversible reduction of reactive chlorine species generated during the chlorine evolution reaction (CER) in the anodic scan at approximately 1.17 V vs Ag/AgCl (1.36 V vs SHE),^12,22,23^ which would otherwise be the main factor responsible for the rapid decrease in the initial OTC concentration, as shown in the inset of Figure 3. In addition, during the exploratory stage (Supporting Information 3), using the electroactive area (5.08 cm^2^), current density values of 6.0, 10, and 20 A m^–2^ were calculated in the factorial design. Note that the *j* selection was carried out in the potential range where the OER contribution was lower.

As previously described, the pollutant elimination efficiency is influenced by Cl_2_-active production. Accordingly, the optimization of pollutant degradation indirectly involves the maximization of chlorine species production. It has been reported that the current density and media pH are the parameters that most influence the production of active chlorine within typical experimental conditions and, consequently, the degradation percentage of emerging pollutants.^20,22−24^ It is worth noting that the chloride concentration is not a parameter that typically varies under environmental conditions since most wastewater contains a fixed concentration of these ions.

**Figure 4:**
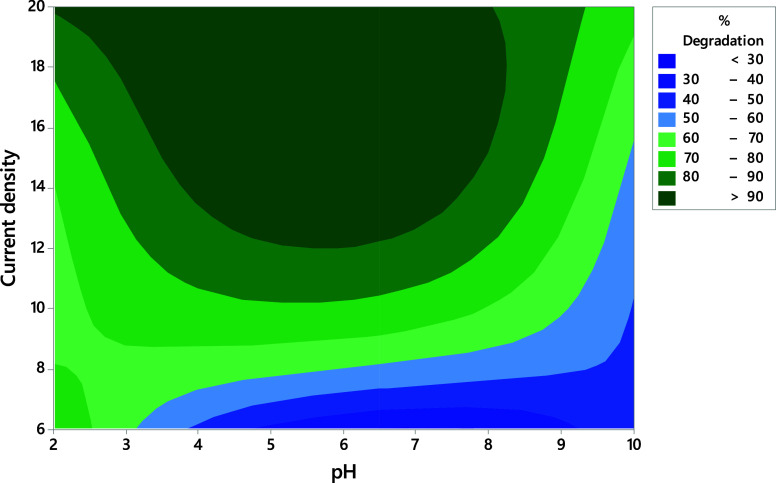
Contour graph showing the percentage of 43.4 μmol L^–1^ OTC degradation from Cl_2_-active electrogenerated due to variations of *j* = 6.0–20 A m^–2^ and pH (3–10).

Figure 4 shows a contour graph where the most intense areas with lower percentages of degradation are highlighted in blue, while the most intense green areas indicate regions where pollutant removal percentages are favored.

**Figure 5:**
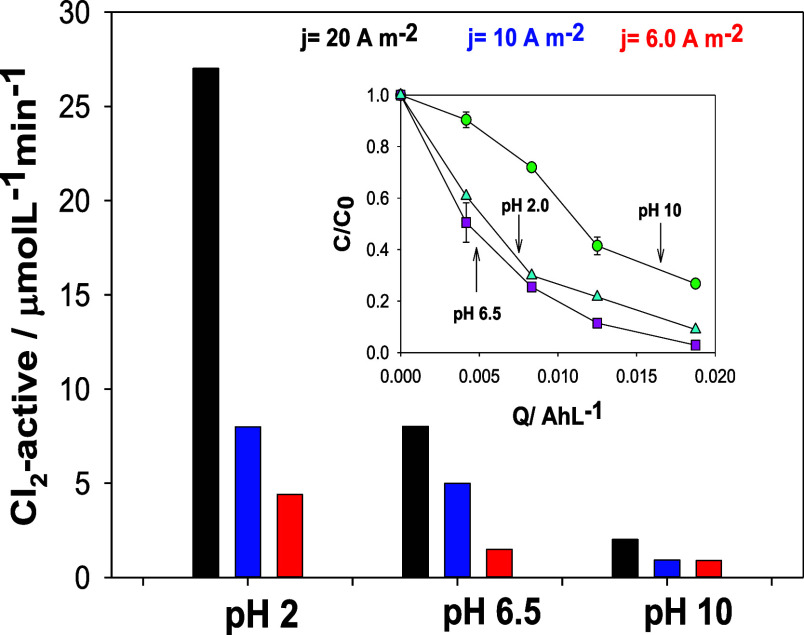
Active chlorine generated during 0.1 mol L^–1^ NaCl electrolysis at different initial current densities (*j* = 20, 10, and 6.0 A m^–2^) and initial pH values (2, 6.5, and 10). Figure inset: pH effect during 43.4 μmol L^–1^ OTC degradation in 0.1 mol L^–1^ NaCl at 20 A m^–2^.

**Figure 6:**
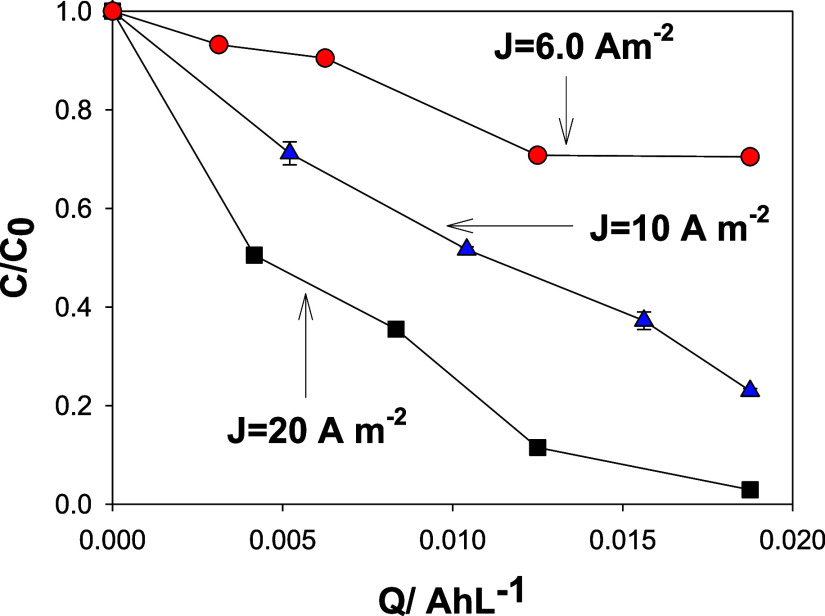
Normalized evolution profiles of OTC concentrations as a function of charge (A h L^–1^). Conditions: 43.4 μmol L^–1^ oxytetracycline, 0.1 mol L^–1^ NaCl, and natural pH (6.5).

It is not surprising to observe in this graph that a higher current density of approximately 20 A m^–2^ increases the percentage of degradation since the quantity of active chlorine produced strongly depends on the current applied to the electrodes. This behavior is shown in Figure 5 during the monitoring of the evolution of the concentration of electrogenerated active chlorine as a function of the charge transferred during electrolysis at 6.0, 10, and 20 A m^–2^ in the absence of oxytetracycline. Therefore, more active chlorine species will form, increasing the initial rate of the removal of OTC once the current density increases in the system. In addition, the initial rate of OTC removal, determined by linearizing the OTC elimination (*C*/*C*_0_) vs the electrolysis time (min), linearly increased with increasing current density, with values of 1.5, 5.0, and 8.0 μmol L^–1^ min^–1^ at 6.0, 10, and 20 A m^–2^, respectively (Figure 6).

In general, the decrease in the OTC concentration was significantly faster at the highest current density, involving a charge transfer of 0.019 A h L^–1^ (Figure 6), and complete elimination of oxytetracycline was achieved at 20 A m^–2^. Precisely within the highest range of current, the widest range of pH values (3–7.8) is located, which can be used to obtain degradation percentages greater than 90%, as shown in Figure 4, even at pH 2, where the oxidant concentration was up to three times greater than the other pH values, but the OTC elimination was similar to that at pH 6.5. This is mainly because the HClO species (HClO/Cl^–^, *E*_redox_^°^ = 1.49 V vs SHE) with the most positive reduction potential is predominantly produced in this pH region compared to other chlorine species typically formed on electrocatalysts, such as Cl_2_ (Cl_2_/Cl^–^, *E*_redox_^°^ = 1.36 V vs SHE acid zone) or OCl^–^ (OCl^–^/Cl^–^, *E*_redox_^°^ = 0.89 V vs SHE, alkaline zone). This information is confirmed by the values obtained for degradation in Table 1. It is also evident from Figure 4 that if the current density is reduced to 11.8 A m^–2^, the pH range that can be used to sustain the percentages of degradation above 80% is between 4.0 and 7.0, indicating that this zone is dominated by HOCl species. It is worth noting that the O_2_ evolution reaction occurs as a parasitic reaction at the anode; therefore, at very high current values, it could exhibit a more important contribution than the reactive species of chloride in the OTC oxidation, decreasing the current efficiency.

The contour plot also reveals that the current density is a more critical parameter influencing the degradation rate than the pH since the zone with a higher degradation rate does not even cover half of the region of the current density values analyzed in the present study; thus, its variation is more important for increasing the production of active chlorine. Similarly, Table 2 shows that the degradation percentages decrease more drastically when the current density is decreased than when the pH is altered. From the optimization process, the following regression equation was obtained, describing the degradation percentage as a function of the design input parameters as a function of encoded variables
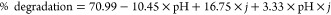
9

**Table 2:** Analysis of Variance (ANOVA) Estimated for the Factorial Design Experiment Described in Table 1 Using the Pollutant Degradation as a Response Variable

source	df	sum of squares	mean square	*F* value	*p* value
model	5	2593.17	518.63	1.29	0.443
pH	1	641.47	641.47	1.60	0.295
current density	1	1732.17	1732.17	4.32	0.129
quadratic	2	8.71	4.35	0.01	0.989
pH × pH	1	1.91	1.91	0.00	0.949
current density × current density	1	6.80	6.80	0.02	0.905
interaction of two factors	1	47.42	47.42	0.12	0.754
pH × current density	1	47.42	47.42	0.12	0.754
error	3	1203.25	401.08		
total	8	3796.42			

Table 2 shows the analysis of variance (ANOVA) of the response regression model (squared surface) based on the percentage of OTC degradation. As observed, the low value obtained for the parameter *p* in the model (0.443) suggested that the previous equation correctly explained the percentage of pollutant degradation. On the other hand, the highest value associated with the column *F* parameter is obtained for the current density (4.32), confirming that it is the most significant parameter influencing the response variable, while the interaction of the pH with the current is not correlated (0.12); consequently, this interaction does not significantly modify the degradation percentage, perhaps since they are independent of each other.

### 3.3 Degradation Mechanism of OTC Abatement by Active Chlorine and Possible Byproducts

**Figure 7:**
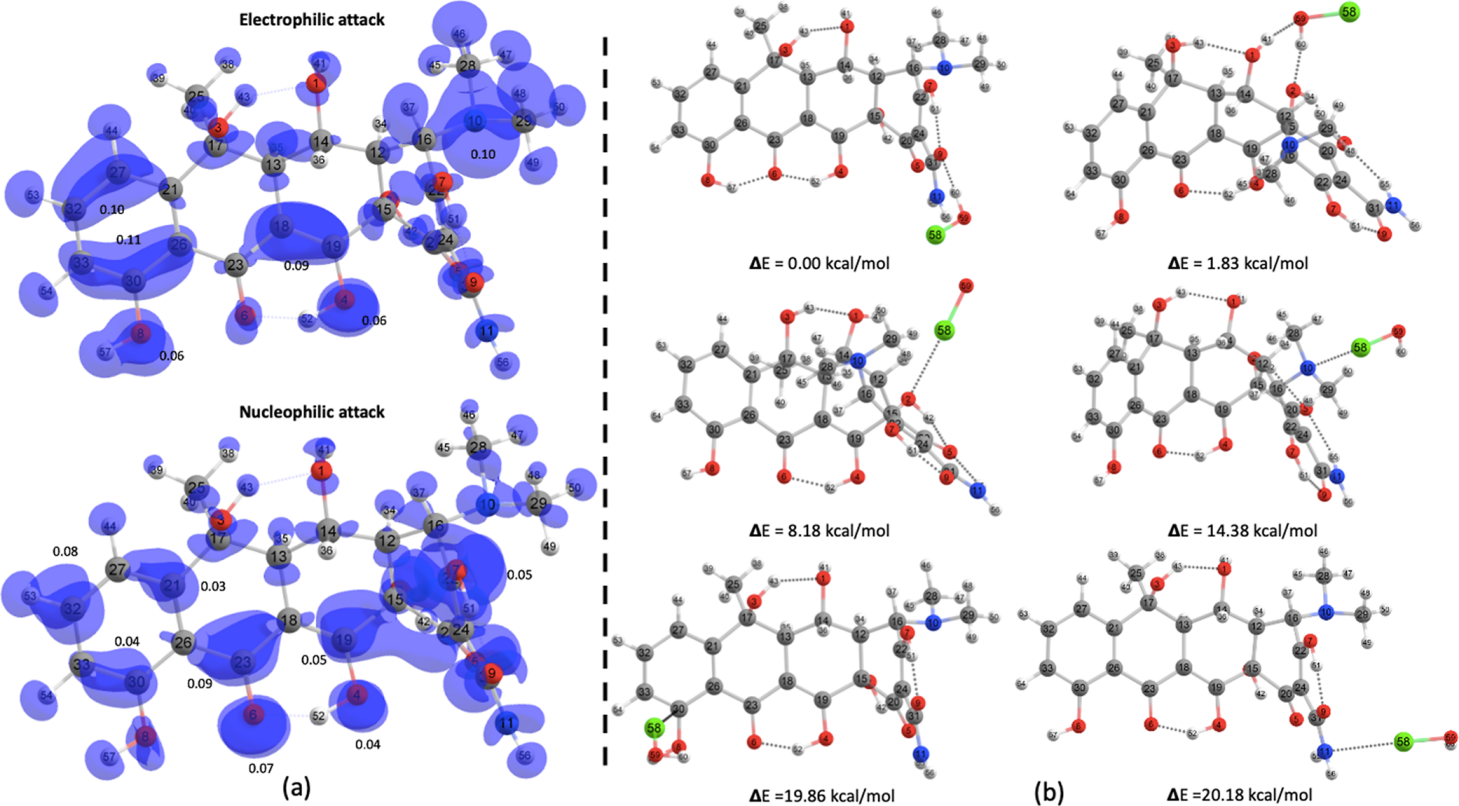
(a) Surface plots displaying condensed nucleophilic and electrophilic Fukui functions computed for the fundamental states (B3LYP/6-31 + g(d)) of OTC. (b) OTC–HOCl PES exploration highlighting the most electronically stable structures.

To understand the mechanism of the effect of HClO (Cl^δ+^–HO^δ−^) species on OTC (C_22_H_24_N_2_O_9_) degradation, quantum chemical analysis was performed to infer the nucleophilic and electrophilic sites favored for attack by reactive chlorine species (Supporting Information 4–6). Thus, a nucleophilic attack highlights the sites within the molecules where the electronic density is lower because it is an electron acceptor, while an electrophilic attack enhances the region by donating electron density. Figure 7 shows the topological analyses for each region of the OTC and the most representative structures in the PES. The analyses suggest that the sites located at the nitrogen, oxygen, and double carbon bond atoms have the highest electron density and are most likely subjected to HClO electrophilic attack.

**Figure 8:**
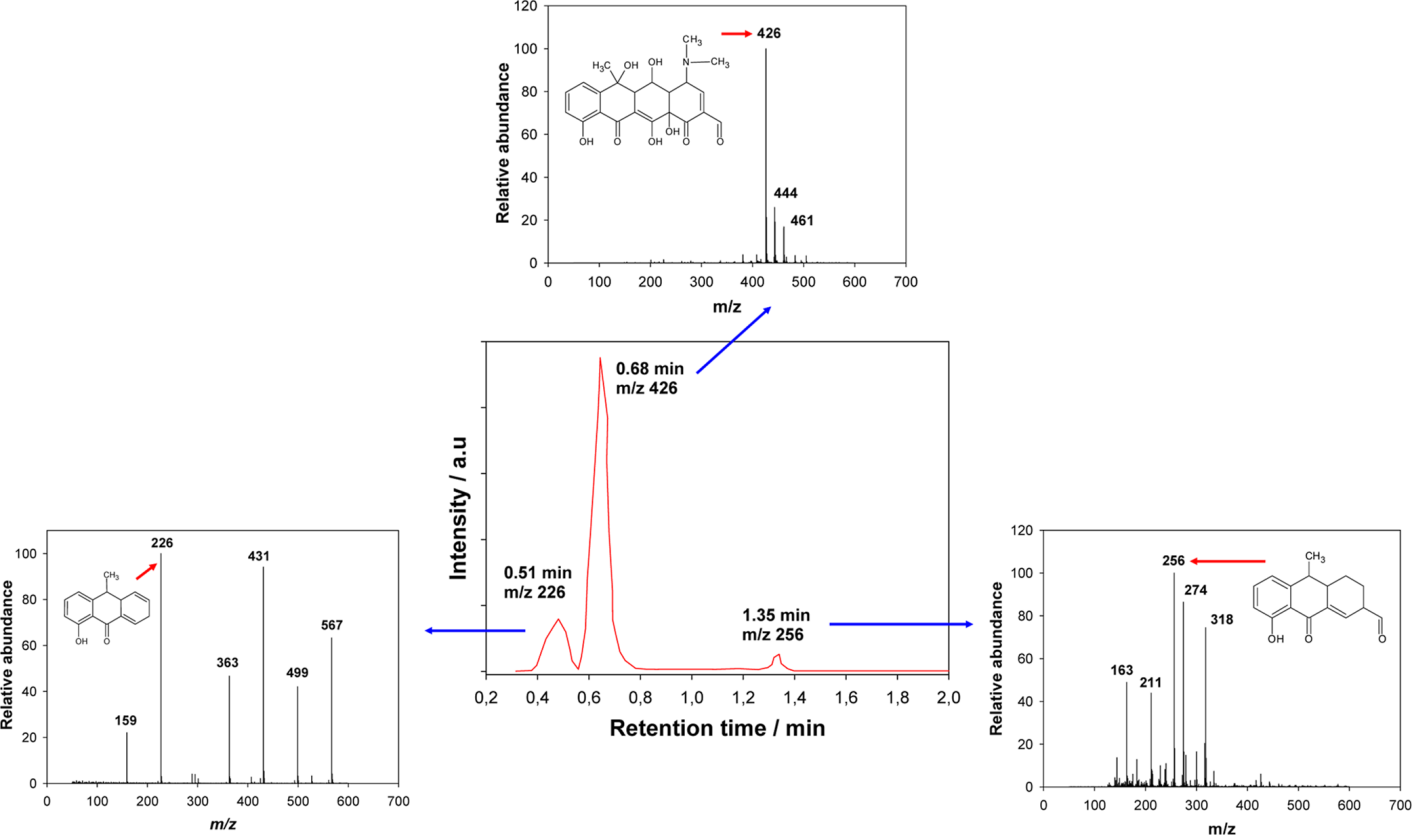
HPLC–MS spectra collected during the electrooxidation of 43.4 μmol L^–1^ OTC in 0.1 mol L^–1^ NaCl at 20 A m^–2^ at 50% degradation time. The insets show mass spectra eluted at peaks of 0.51, 0.68, and 1.35 min.

These results were confirmed through a PES exploration between OTC and HOCl to form the OTC–HOCl adduct (see Figure 7 and Supporting Information 8). In this context, the interaction between HO–Cl–OH leads to a dehydroxylation procedure (involving the OTC oxygen atoms); the N–Cl–OH interaction facilitates deamination (at N11) and demethylation (at N10), notably resulting in the breakage of the C16–N10 bond and the formation of a dimethylamine molecule as well as aromatic ring chlorination. Similar results were reported for oxytetracycline degradation in water by different oxidants,^25−27^ which are mainly involved in hydroxylation, dehydroxylation, decarbonylation, demethylation, hydrogen extraction, and deamination.^28−32^ Additionally, these results concerning OTC oxidation were consistent with the reported reactivity of chlorine species such as hypochlorous acid, which preferentially attacks activated amides, phenols, and reduced moieties on the structure of organic pollutants.^33^ In addition to the theoretical calculations, chromatographic detection of some primary stable transformation byproducts was performed. Figure 8 shows the chromatogram for the OTC after 8.6 min of electrolysis using 20 A m^–2^. OTC reacted rapidly with HOCl, and less than 1% of the initial level of OTC was detected after 10 min of reaction initiation. The results showed that the electrochemical process produced three main degradation byproducts (C_22_H_23_NO_8_, OTC-I-1; C_16_H_16_O_3_, OTC-I-2; and C_15_H_14_O_2_, OTC-I-3). Similar reports regarding chlorination and monochloramination of oxytetracycline have reported that byproducts such as C_22_H_23_NO_9_ and C_22_H_21_NO_8_ are easily lost due to chlorine substitution reactions, followed by dehydration or oxidation to the *N*-methyl and amino groups from OTC because of the low bond energy of N–C,^25−27,34,35^ which was consistent with the Fukui analysis. In addition, other pathways involving hydroxylation (C_22_H_26_NO_11_) and Cl substitution (C_22_H_24_N_2_O_9_Cl) have been proposed.^34−36^ However, these byproducts were not detected. Regarding the last possible byproducts, electrophilic aromatic substitution will occur readily with many aromatic derivatives and polycyclic aromatic compounds, such as those produced by oxytetracycline, and can be activated to interact with electrogenerated HOCl. On the other hand, the presence of trihalomethane compounds was not detected in the HPLC–MS measurements above-described according to different *m*/*z* ratios reported in the literature for chlorine-based oxidants (e.g., chloroform).^37,38^ This is not surprising since THMs arise from reactions between oxidants (i.e., free chlorine) and natural organic matter (i.e., humic acid), which is not present in the synthetic solution evaluated in this study.

**Figure 9:**
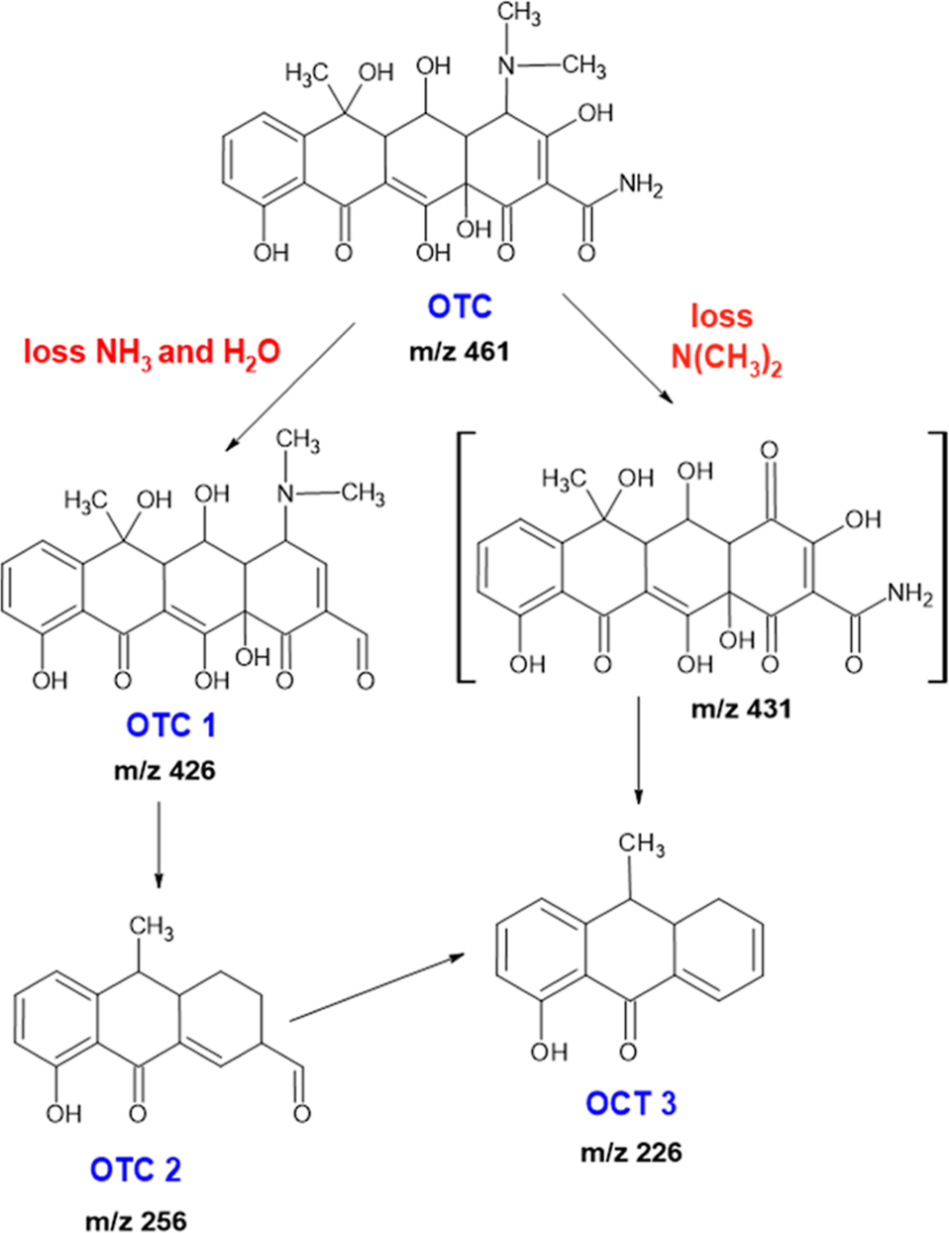
Proposed degradation mechanism of OTC during electrochemical degradation mediated by chlorides.

Figure 9 shows the possible mechanism of degradation of oxytetracycline by active chlorine. In the first stage, the active chlorine species attacks the susceptible site located in the amide group with subsequent loss of the amine of C31 and removal of the hydroxyl group of C22, generating the byproduct, OTC-I-1 with *m*/*z* 426. In addition, the attack of the amine ring was carried out based on a hydrogen abstraction mechanism. Further chlorine-active species attack would drive the occurrence of C–C bond cleavage, producing carbonylic compounds and amines, as indicated by the lack of detection of the byproduct at *m*/*z* 431. Simultaneously, aromatic electrophilic substitution was carried out by the addition of chlorine and hydroxyl ions to the phenolic ring of oxytetracycline. This could lead to successive oxidation, resulting in the formation of short-chain derivatives lacking antibiotic activity (Supporting Information 7).

## 4 Conclusions

A Ti/TiO_2_–RuO_2_ anode material with excellent electrocatalytic performance for the degradation of the OTC was successfully prepared by the Pechini method. SEM images and XRD data demonstrated that Ti/TiO_2_–RuO_2_ consisted of a solid solution formed by RuO_2_ rutile and TiO_2_ rutile (*P*4/*mmm*). Within 20 min, an approximately 97% removal rate of the OTC was achieved. CV showed that Ti/TiO_2_–RuO_2_ mainly degraded OTC by electrogenerated active chlorine species without direct oxidation pathway participation. Based on the experimental design, the best degradation rates were obtained at a high current density and a pH of nearly 6.0, although the energy consumption was moderately high. Under the best conditions (20 A m^–2^, pH 6.5, and 0.1 mol/L NaCl), the phosphorus of OTC and its aromatic byproducts were efficiently removed. Quantum chemical calculations using DFT were combined with HPLC results to propose a reaction pathway for OTC degradation, and to identify the active sites of OTC that were attacked by active chlorine species. Three intermediates were detected during OTC abatement, and this transformation involved the deamination, Cl-substitution, and hydroxylation of unsaturated double bonds on aromatic rings, amines, and amides. The presence of trihalomethane compounds was not detected in the HPLC–MS measurements.

## Supporting Information Available

TGA and DTA of precursor solution of RuO_2_; electrochemical system for the anodic oxidation of oxytetracycline; kinetics of the Fe (CN)_6_^3–^/Fe (CN)_6_^4–^ couple occurring on DSA described by CV; Hirshfeld charges, condensed Fukui functions, and condensed dual descriptors; condensed local electrophilicity/nucleophilicity index, condensed local softnesses relative electrophilicity/nucleophilicity and condensed local hyper-softness; representative results of the antibiotic activity test using *Staphylococcus aureus* at 0, 15, 30, 60, and 120 min of electrochemical treatment; and OTC–HOCl PES exploration results (PDF)
