# Supplementary material for: Reaction Mechanism of Oxytetracycline Degradation by Electrogenerated Reactive Chlorine: The Influence of Current Density and pH
Source: ACS Omega. 2024 Nov 6;9(46):46302–11. doi: 10.1021/acsomega.4c07234 (PMC11579939; doi:10.1021/acsomega.4c07234)
Supplement: Supplementary file 1 — ao4c07234_si_001.pdf [file ao4c07234_si_001.pdf]

The reaction mechanism of oxytetracycline degradation by electrogenerated reactive chlorine: the influence of current density and pH

Stephanie Sanchez-Castrillon [a], Luis Norberto Benítez [a], Jorge Vázquez-Arenas [b, *], Franklin Ferraro [c], Ricardo E. Palma-Goyes [a,*]

[a] Departamento de Química, Universidad del Valle, Santiago de Cali, Calle 13 # 100-00, CP 760032, Colombia

[b] Centro Mexicano para la Producción más Limpia, Instituto Politécnico Nacional, Av. Acueducto s/n, Col. La Laguna Ticomán, Ciudad de México 07340, México

[c] Departamento de Ciencias Básicas, Universidad Católica Luis Amigó, Transversal, 51A, #67B 90, Medellín, Colombia

Figure S1. TGA and DTA analysis of precursor solution of RuO_2

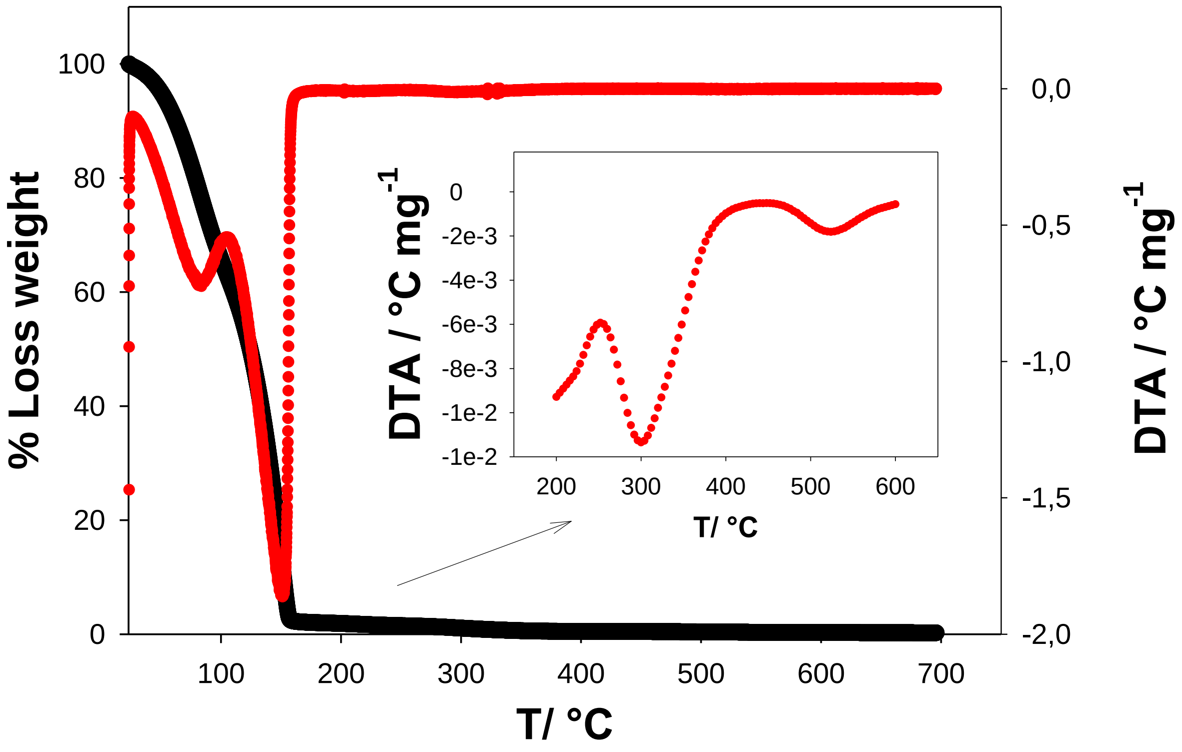

Figure S2. Electrochemical system for anodic oxidation of Oxytetracycline.

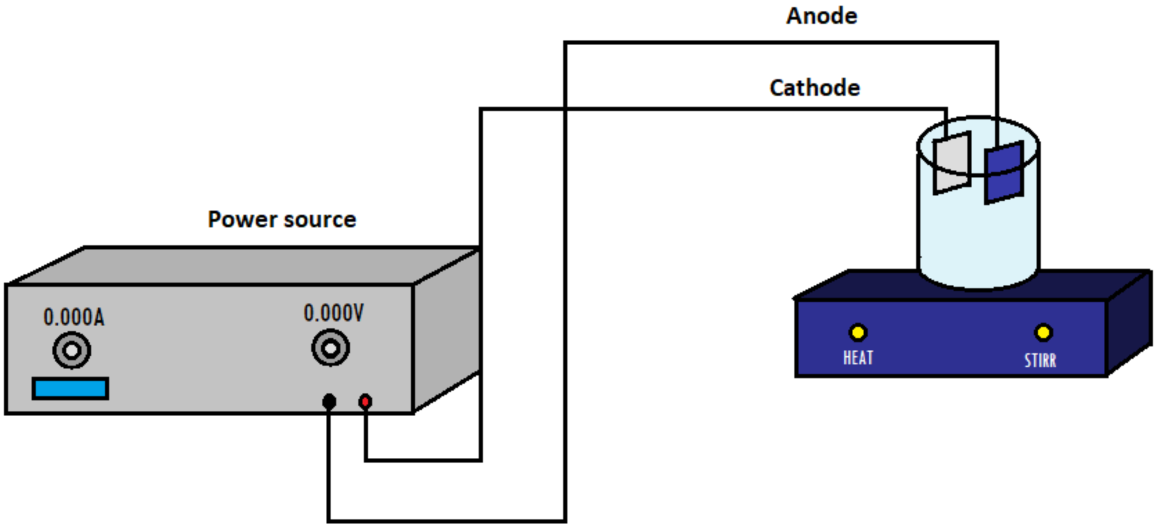

Figure S3. Kinetics of the Fe $(CN)_6^{3-}$/Fe $(CN)_6^{4-}$ couple occurring on DSA describing by cyclic voltammetry

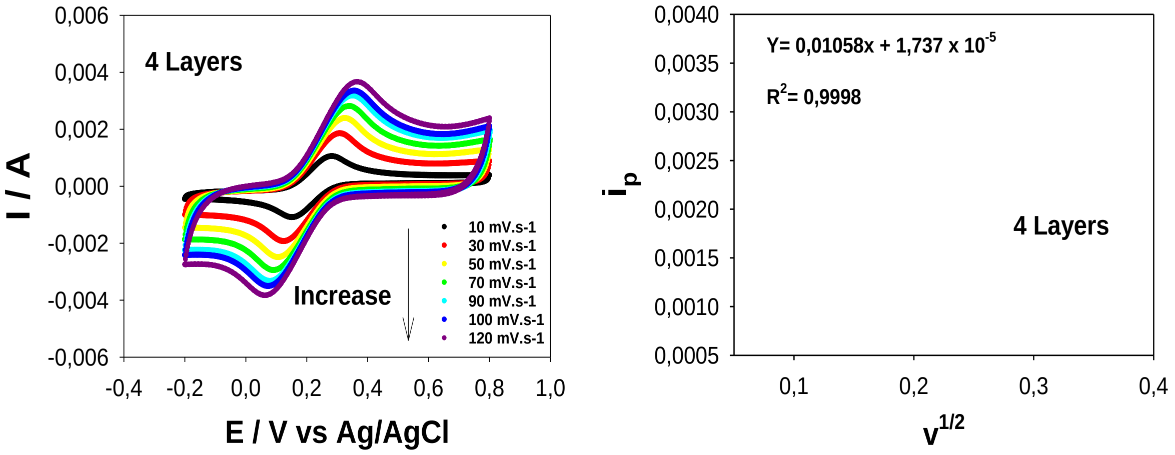

$$Fe(CN)_6^{-3} + e^- \leftrightarrow Fe(CN)_6^{-4}$$

$$i_p = 2.69 \times 10^5 n^{\frac{3}{2}} A D^{\frac{1}{2}} C v^{\frac{1}{2}}$$

$$A = \frac{0.01508 \frac{A\, s^{1/2}}{V^{1/2}}}{2.69 \times 10^5\, n^{\frac{3}{2}}\, D^{\frac{1}{2}}\, C},\ n = 1, C = 0.004\ molcm^3, D = 0.00194 cm^2 s^{-1}$$

Table S1. Hirshfeld charges, condensed Fukui functions and condensed dual descriptors. Units used below are "e" (elementary charge).

Atom	q(N)	q(N+1)	q(N-1)	f-	f+	f0	CDD
1(O)	-0.1926	-0.2042	-0.1790	0.0136	0.0116	0.0126	-0.0020
2(O)	-0.2254	-0.2502	-0.2066	0.0188	0.0248	0.0218	0.0060
3(O)	-0.2334	-0.2413	-0.2209	0.0125	0.0079	0.0102	-0.0046
4(O)	-0.1765	-0.2156	-0.1278	0.0487	0.0391	0.0439	-0.0096
5(O)	-0.2326	-0.2834	-0.2142	0.0184	0.0509	0.0346	0.0325
6(O)	-0.2294	-0.2945	-0.2054	0.0239	0.0652	0.0445	0.0412
7(O)	-0.1522	-0.1926	-0.1421	0.0101	0.0404	0.0253	0.0304
8(O)	-0.1675	-0.1847	-0.1126	0.0549	0.0172	0.0361	-0.0377
9(O)	-0.2875	-0.3136	-0.2705	0.0170	0.0260	0.0215	0.0090
10(N)	-0.0959	-0.0961	-0.0171	0.0788	0.0002	0.0395	-0.0786
11(N)	-0.1232	-0.1374	-0.1141	0.0090	0.0142	0.0116	0.0052
12(C)	-0.0273	-0.0288	-0.0259	0.0014	0.0014	0.0014	0.0000
13(C)	-0.0308	-0.0325	-0.0246	0.0062	0.0017	0.0039	-0.0045
14(C)	0.0539	0.0527	0.0579	0.0040	0.0012	0.0026	-0.0028
15(C)	0.0728	0.0648	0.0732	0.0004	0.0080	0.0042	0.0076
16(C)	0.0202	0.0163	0.0291	0.0089	0.0039	0.0064	-0.0050
17(C)	0.0922	0.0916	0.0931	0.0009	0.0006	0.0008	-0.0003
18(C)	-0.0614	-0.0773	-0.0125	0.0488	0.0160	0.0324	-0.0329
19(C)	0.0981	0.0560	0.1289	0.0308	0.0421	0.0364	0.0113
20(C)	0.1393	0.1055	0.1353	0.0040	0.0338	0.0149	0.0378
21(C)	0.0137	-0.0087	0.0244	0.0107	0.0225	0.0166	0.0118
22(C)	0.1306	0.0846	0.1255	0.0051	0.0460	0.0205	0.0511
23(C)	0.1296	0.0680	0.1391	0.0094	0.0616	0.0355	0.0522
24(C)	-0.0710	-0.0825	-0.0682	0.0027	0.0116	0.0072	0.0088
25(C)	-0.0945	-0.1013	-0.0877	0.0068	0.0068	0.0068	0.0000
26(C)	-0.0289	-0.0393	-0.0030	0.0259	0.0104	0.0181	-0.0155
27(C)	-0.0491	-0.0695	0.0003	0.0494	0.0204	0.0349	-0.0290
28(C)	-0.0392	-0.0461	-0.0225	0.0167	0.0069	0.0118	-0.0097
29(C)	-0.0443	-0.0495	-0.0255	0.0188	0.0051	0.0120	-0.0137
30(C)	0.0924	0.0693	0.1297	0.0374	0.0231	0.0302	-0.0143
31(C)	0.1666	0.1572	0.1691	0.0025	0.0093	0.0059	0.0068
32(C)	-0.0253	-0.0767	0.0109	0.0362	0.0514	0.0438	0.0152
33(C)	-0.0684	-0.0952	-0.0345	0.0339	0.0268	0.0304	-0.0071
34(H)	0.0325	0.0224	0.0375	0.0050	0.0101	0.0075	0.0051
35(H)	0.0281	0.0183	0.0425	0.0144	0.0098	0.0121	-0.0045

36(H)	0.0350	0.0325	0.0416	0.0066	0.0025	0.0046	-0.0041
37(H)	0.0274	0.0171	0.0461	0.0187	0.0103	0.0145	-0.0084
38(H)	0.0234	0.0122	0.0336	0.0102	0.0112	0.0107	0.0009
39(H)	0.0331	0.0223	0.0434	0.0103	0.0108	0.0105	0.0004
40(H)	0.0338	0.0271	0.0410	0.0072	0.0067	0.0070	-0.0005
41(H)	0.1584	0.1482	0.1669	0.0086	0.0102	0.0094	0.0016
42(H)	0.1403	0.1248	0.1521	0.0118	0.0155	0.0137	0.0036
43(H)	0.1259	0.1170	0.1354	0.0095	0.0090	0.0092	-0.0006
44(H)	0.0426	0.0265	0.0636	0.0210	0.0161	0.0185	-0.0048
45(H)	0.0329	0.0291	0.0446	0.0118	0.0038	0.0078	-0.0080
46(H)	0.0201	0.0089	0.0482	0.0281	0.0112	0.0197	-0.0170
47(H)	0.0409	0.0278	0.0588	0.0179	0.0130	0.0155	-0.0049
48(H)	0.0210	0.0103	0.0506	0.0295	0.0108	0.0202	-0.0187
49(H)	0.0307	0.0290	0.0450	0.0143	0.0018	0.0080	-0.0125
50(H)	0.0387	0.0256	0.0566	0.0179	0.0131	0.0155	-0.0048
51(H)	0.1356	0.1225	0.1425	0.0069	0.0131	0.0100	0.0062
52(H)	0.1241	0.1084	0.1374	0.0133	0.0157	0.0145	0.0024
53(H)	0.0500	0.0207	0.0738	0.0238	0.0293	0.0265	0.0055
54(H)	0.0426	0.0215	0.0652	0.0227	0.0211	0.0219	-0.0016
55(H)	0.1165	0.1077	0.1208	0.0043	0.0088	0.0066	0.0045
56(H)	0.1401	0.1214	0.1519	0.0118	0.0187	0.0152	0.0069
57(H)	0.1736	0.1550	0.1993	0.0257	0.0186	0.0221	-0.0070

Table S2. Condensed local electrophilicity/nucleophilicity index (e*eV)

Atom	Electrophilicity	Nucleophilici
1(O)	0.01559	0.03663
2(O)	0.03334	0.05079
3(O)	0.01060	0.03366
4(O)	0.05258	0.13149
5(O)	0.06838	0.04965
6(O)	0.08758	0.06457
7(O)	0.05435	0.02718
8(O)	0.02318	0.14822
9(O)	0.03501	0.04591
10(N)	0.00026	0.21260
11(N)	0.01915	0.02433
12(C)	0.00193	0.00383
13(C)	0.00225	0.01679
14(C)	0.00163	0.01089
15(C)	0.01078	0.00110
16(C)	0.00526	0.02393
17(C)	0.00082	0.00250
18(C)	0.02145	0.13175
19(C)	0.05656	0.08307
20(C)	0.04542	-0.01078
21(C)	0.03020	0.02883
22(C)	0.06185	-0.01372
23(C)	0.08285	0.02543
24(C)	0.01557	0.00739
25(C)	0.00915	0.01827
26(C)	0.01396	0.06984
27(C)	0.02747	0.13327
28(C)	0.00931	0.04494
29(C)	0.00687	0.05076
30(C)	0.03106	0.10088
31(C)	0.01256	0.00675
32(C)	0.06911	0.09760
33(C)	0.03602	0.09154
34(H)	0.01355	0.01351
35(H)	0.01324	0.03882

36(H)	0.00339	0.01779
37(H)	0.01389	0.05043
38(H)	0.01501	0.02762
39(H)	0.01446	0.02789
40(H)	0.00900	0.01950
41(H)	0.01367	0.02310
42(H)	0.02080	0.03193
43(H)	0.01204	0.02567
44(H)	0.02170	0.05653
45(H)	0.00511	0.03172
46(H)	0.01503	0.07591
47(H)	0.01754	0.04843
48(H)	0.01450	0.07965
49(H)	0.00235	0.03851
50(H)	0.01765	0.04836
51(H)	0.01757	0.01851
52(H)	0.02111	0.03579
53(H)	0.03935	0.06424
54(H)	0.02835	0.06112
55(H)	0.01183	0.01162
56(H)	0.02514	0.03181
57(H)	0.02502	0.06922

Table S3. Condensed local softnesses (Hartree*e), relative electrophilicity/nucleophilicity (dimensionless) and condensed local hyper-softness (e/Hartree^2).

Atom	s-	s+	s0	s+/s-	s-/s+	s(2)
1(O)	0.0538	0.0460	0.0499	0.8544	1.1704	-0.0310
2(O)	0.0746	0.0983	0.0865	1.3176	0.7590	0.0939
3(O)	0.0494	0.0312	0.0403	0.6320	1.5822	-0.0721
4(O)	0.1931	0.1550	0.1741	0.8026	1.2459	-0.1511
5(O)	0.0729	0.2016	0.1373	2.7645	0.3617	0.5101
6(O)	0.0948	0.2582	0.1765	2.7227	0.3673	0.6476
7(O)	0.0399	0.1603	0.1001	4.0149	0.2491	0.4770
8(O)	0.2177	0.0683	0.1430	0.3139	3.1857	-0.5920
9(O)	0.0674	0.1032	0.0853	1.5309	0.6532	0.1419
10(N)	0.3123	0.0008	0.1565	0.0025	404.0088	-12.347
11(N)	0.0357	0.0565	0.0461	1.5798	0.6330	0.0821
12(C)	0.0056	0.0057	0.0057	1.0129	0.9872	0.0003
13(C)	0.0247	0.0066	0.0156	0.2688	3.7197	-0.0715
14(C)	0.0160	0.0048	0.0104	0.3006	3.3267	-0.0443
15(C)	0.0016	0.0318	0.0167	197.399	0.0507	0.1196
16(C)	0.0352	0.0155	0.0253	0.4411	2.2670	-0.0779
17(C)	0.0037	0.0024	0.0030	0.6536	1.5300	-0.0051
18(C)	0.1935	0.0632	0.1284	0.3268	3.0602	-0.5164
19(C)	0.1220	0.1668	0.1444	1.3668	0.7316	0.1774
20(C)	-0.0158	0.1339	0.0590	-84.596	-0.1182	0.5936
21(C)	0.0423	0.0891	0.0657	2.1028	0.4755	0.1851
22(C)	-0.0202	0.1824	0.0811	-90.489	-0.1105	0.8027
23(C)	0.0374	0.2443	0.1408	6.5396	0.1529	0.8202
24(C)	0.0108	0.0459	0.0284	4.2313	0.2363	0.1389
25(C)	0.0268	0.0270	0.0269	1.0049	0.9952	0.0005
26(C)	0.1026	0.0412	0.0719	0.4014	2.4914	-0.2434
27(C)	0.1958	0.0810	0.1384	0.4137	2.4171	-0.4549
28(C)	0.0660	0.0275	0.0467	0.4161	2.4036	-0.1528
29(C)	0.0746	0.0203	0.0474	0.2717	3.6800	-0.2152
30(C)	0.1482	0.0916	0.1199	0.6182	1.6177	-0.2243
31(C)	0.0099	0.0370	0.0235	3.7350	0.2677	0.1075
32(C)	0.1434	0.2038	0.1736	1.4214	0.7035	0.2395
33(C)	0.1345	0.1062	0.1203	0.7898	1.2662	-0.1120
34(H)	0.0198	0.0399	0.0299	2.0136	0.4966	0.0797
35(H)	0.0570	0.0390	0.0480	0.6845	1.4610	-0.0713

36(H)	0.0261	0.0100	0.0181	0.3825	2.6143	-0.0640
37(H)	0.0741	0.0409	0.0575	0.5527	1.8093	-0.1313
38(H)	0.0406	0.0443	0.0424	1.0913	0.9163	0.0147
39(H)	0.0410	0.0426	0.0418	1.0404	0.9611	0.0066
40(H)	0.0286	0.0265	0.0276	0.9267	1.0791	-0.0083
41(H)	0.0339	0.0403	0.0371	1.1879	0.8418	0.0253
42(H)	0.0469	0.0613	0.0541	1.3078	0.7646	0.0572
43(H)	0.0377	0.0355	0.0366	0.9417	1.0620	-0.0087
44(H)	0.0830	0.0640	0.0735	0.7705	1.2978	-0.0755
45(H)	0.0466	0.0151	0.0308	0.3236	3.0901	-0.1249
46(H)	0.1115	0.0443	0.0779	0.3974	2.5166	-0.2663
47(H)	0.0711	0.0517	0.0614	0.7269	1.3757	-0.0770
48(H)	0.1170	0.0427	0.0799	0.3653	2.7373	-0.2943
49(H)	0.0566	0.0069	0.0318	0.1227	8.1509	-0.1967
50(H)	0.0710	0.0521	0.0615	0.7327	1.3648	-0.0753
51(H)	0.0272	0.0518	0.0395	1.9057	0.5248	0.0976
52(H)	0.0526	0.0623	0.0574	1.1840	0.8446	0.0383
53(H)	0.0944	0.1160	0.1052	1.2296	0.8132	0.0859
54(H)	0.0898	0.0836	0.0867	0.9313	1.0738	-0.0245
55(H)	0.0171	0.0349	0.0260	2.0432	0.4894	0.0706
56(H)	0.0467	0.0741	0.0604	1.5865	0.6303	0.1086
57(H)	0.1017	0.0738	0.0877	0.7257	1.3780	-0.1106

Figure S4. Representative results of antibiotic activity test using S. aureus at 0, 15, 30, 60 and 120 minutes of electrochemical treatment.

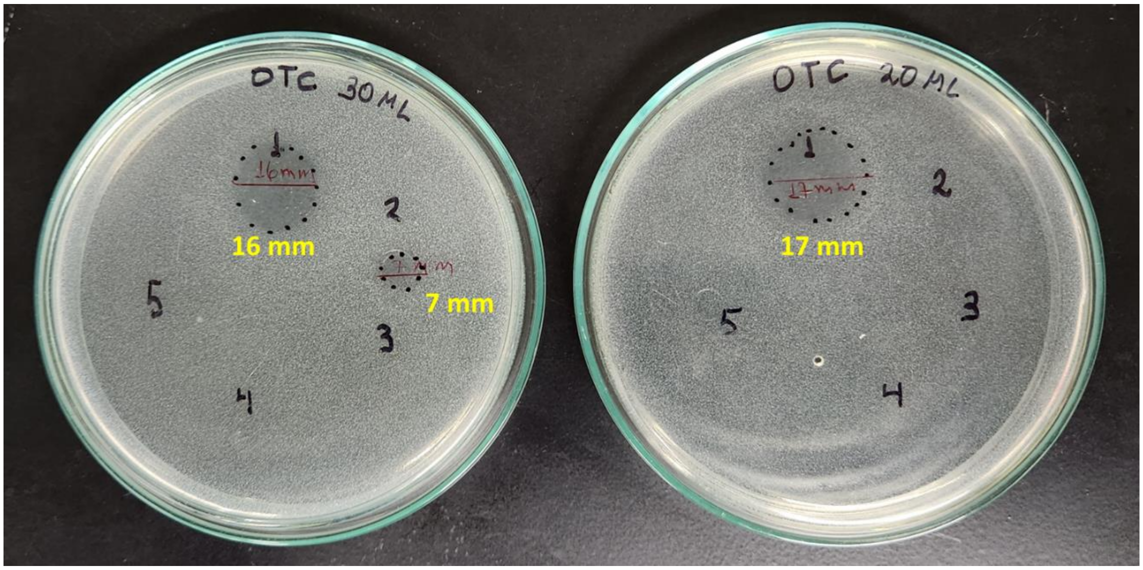

Figure S5. OTC-HOCl PES exploration results. The relative energy is in kcal/mol.

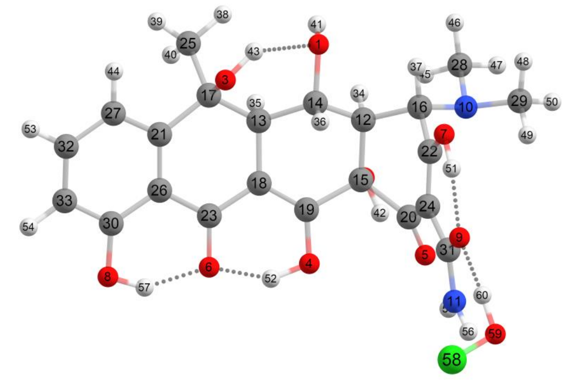	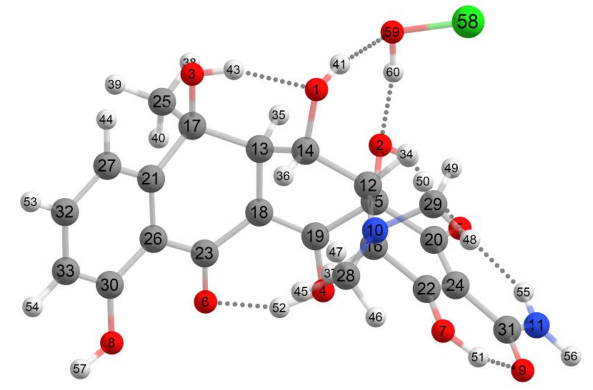
**Δ**E = 0.00 kcal/mol	**Δ**E = 1.83 kcal/mol
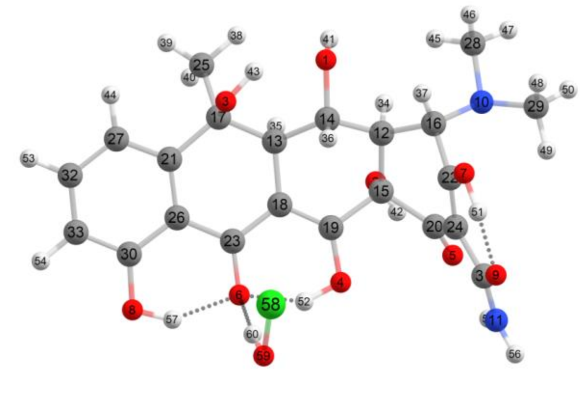	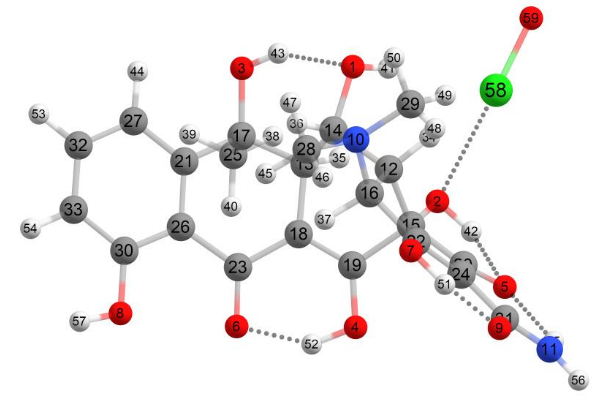
**Δ**E = 4.97 kcal/mol	**Δ**E = 8.18 kcal/mol
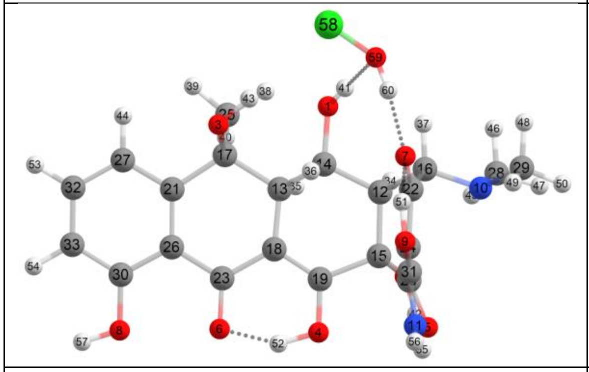	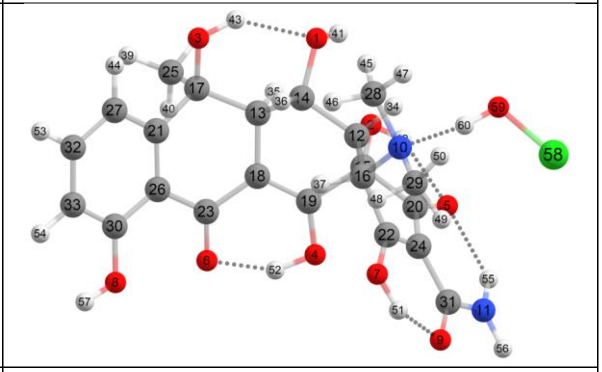
**Δ**E = 9.34 kcal/mol	**Δ**E = 10.48 kcal/mol

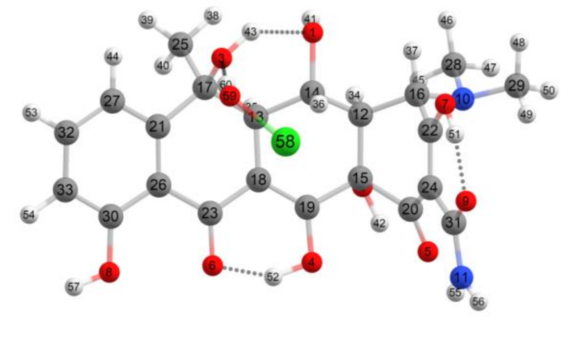	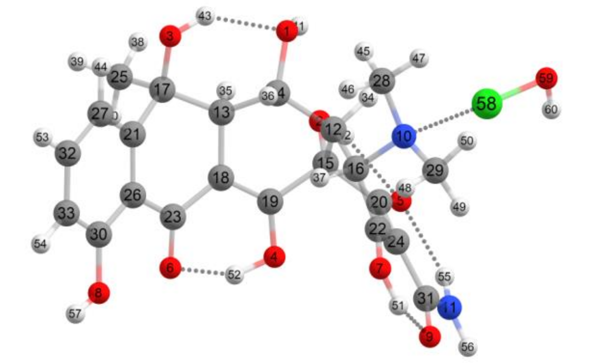
**Δ**E = 13.19 kcal/mol	**Δ**E = 14.38 kcal/mol
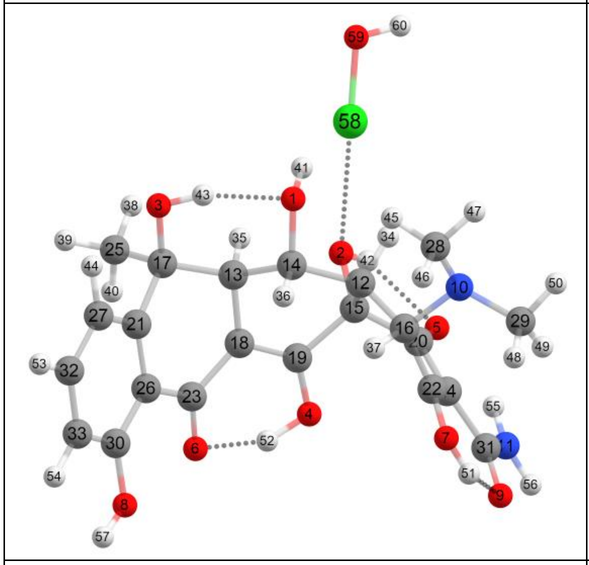	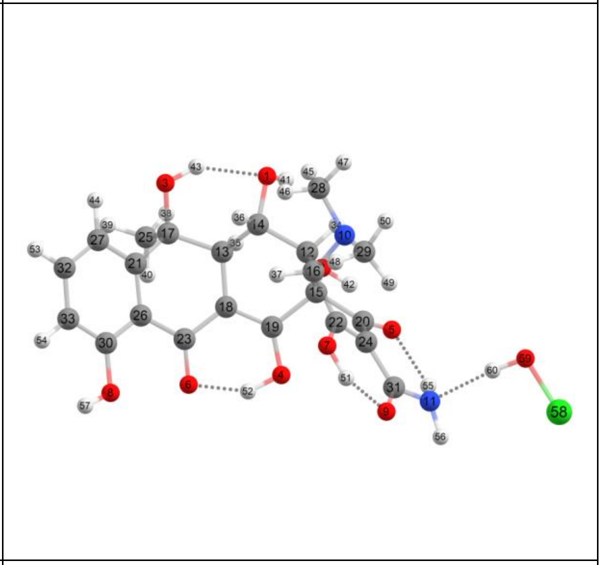
**Δ**E = 16.68 kcal/mol	**Δ**E = 16.80 kcal/mol
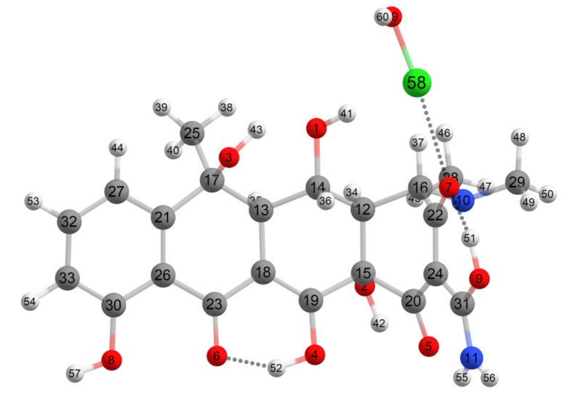	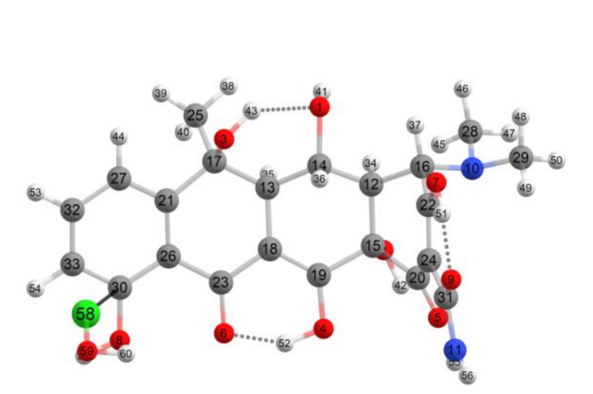
**Δ**E = 17.03 kcal/mol	**Δ**E = 19.86 kcal/mol

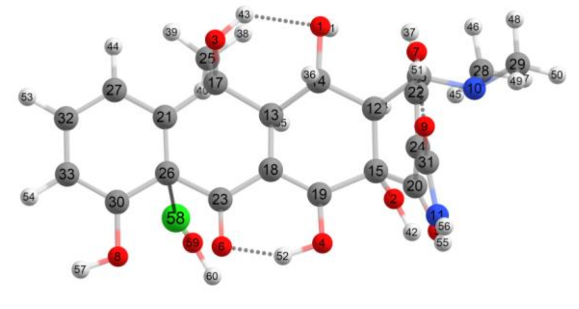	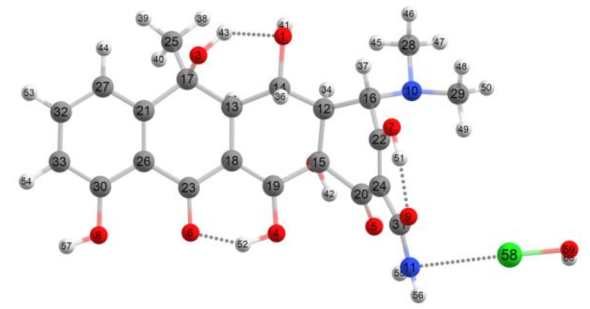
**Δ**E = 19.88 kcal/mol	**Δ**E = 20.18 kcal/mol
